# A novel dedicated nonslip short-length balloon catheter for treating hepaticojejunostomy anastomotic stricture in balloon enteroscopy-assisted ERCP

**DOI:** 10.1055/a-2218-3193

**Published:** 2023-12-21

**Authors:** Tadahisa Inoue, Mayu Ibusuki, Rena Kitano, Kiyoaki Ito

**Affiliations:** 1 12703 Department of Gastroenterology, Aichi Medical University, Nagakute, Japan

Hepaticojejunostomy anastomotic strictures (HJASs) often occur as an adverse event after biliary reconstructive surgery 1 . Balloon stricture dilation via balloon enteroscopy-assisted endoscopic retrograde cholangiopancreatography (BE-ERCP) is a standard treatment for HJAS 2 ; however, there is no dedicated balloon catheter for HJAS treatment. Conventional papillary/bile duct dilation balloons are generally used, but they are often too long for HJASs. Long balloons can unnecessarily expand the normal intrahepatic bile duct and require there to be sufficient distance between the anastomotic site and the tip of the scope, which can be challenging to achieve. In contrast, short balloons tend to slip during inflation, especially during BE-ERCP because of the absence of a forceps elevator.

To address these problems, a novel, dedicated balloon catheter (RIGEL Balloon Dilatation Catheter; Japan Lifeline Co., Ltd., Tokyo, Japan) has been developed ( Fig. 1 ), which has a very short balloon, measuring 15 mm, and an elastic band attached in the middle. The central part expands with a delay, preventing slippage.

**Fig. 1:**
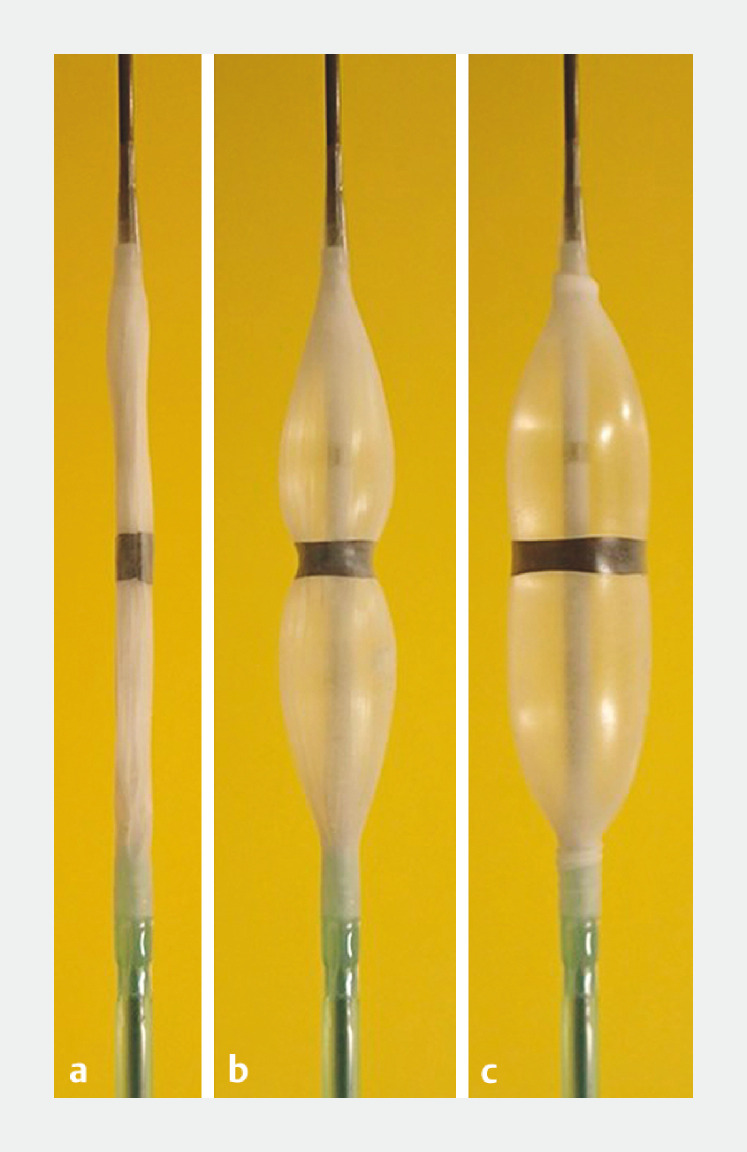
Photographs of the novel dedicated balloon catheter, which has: **a** a very short length of 15 mm; **b,c** a 5-mm elastic ring-band at the center, which means the central part of the balloon expands with a delay during inflation, thereby preventing slippage.

An 88-year-old woman who had undergone pancreaticoduodenectomy developed cholangitis. A short-type single-balloon enteroscope was inserted, and the HJAS was detected. After inserting a guidewire through the stricture, we inserted the 8-mm diameter novel balloon catheter over the guidewire. The central part of the novel balloon expanded with an appropriate delay during inflation, achieving full expansion without slippage, while maintaining a position close to the anastomosis ( Fig. 2 ; Video 1 ). The stricture was finally well recanalized, and no adverse events occurred.

**Fig. 2:**
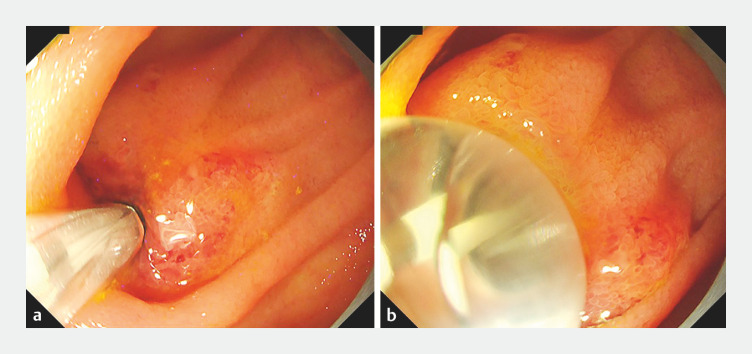
Endoscopic images showing: **a** the novel balloon inserted and positioned with its center at the stricture after biliary cannulation through the hepaticojejunostomy anastomotic stricture had been performed during short-type single-balloon enteroscopy; **b** expansion of the center of the novel balloon after an appropriate delay, which allows full expansion without slippage, while maintaining a position close to the anastomosis.

Treatment of a hepaticojejunostomy anastomotic stricture using a novel dedicated nonslip balloon catheter of 8-mm diameter during balloon enteroscopy-assisted endoscopic retrograde cholangiopancreatography.Video 1

This novel balloon catheter offers a new device option for BE-ERCP. Its unique short length and antislip features make it suitable for the treatment of HJASs.

Endoscopy_UCTN_Code_TTT_1AP_2AD

## Notes

Endoscopy E-Videos https://eref.thieme.de/e-videos
*E-Videos* is an open access online section of the journal *Endoscopy* , reporting on interesting cases and new techniques in gastroenterological endoscopy. All papers include a high-quality video and are published with a Creative Commons CC-BY license. Endoscopy E-Videos qualify for HINARI discounts and waivers and eligibility is automatically checked during the submission process. We grant 100% waivers to articles whose corresponding authors are based in Group A countries and 50% waivers to those who are based in Group B countries as classified by Research4Life (see: https://www.research4life.org/access/eligibility/ ). This section has its own submission website at https://mc.manuscriptcentral.com/e-videos .
